# Effect of high-power-laser with and without graphite coating on bonding of resin cement to lithium disilicate ceramic

**DOI:** 10.1038/s41598-017-17808-x

**Published:** 2017-12-12

**Authors:** Fernanda A. Feitosa, Rodrigo M. de Araújo, Franklin R. Tay, Lina Niu, César R. Pucci

**Affiliations:** 1 Department of Restorative Dentistry, Institute of Science and Technology, São Paulo State University UNESP São Jose dos Campos, São Paulo, Brazil; 2 Department of Dental Materials and Prosthodontics, Institute of Science and Technology, São Paulo State University UNESP São Jose dos Campos, São Paulo, Brazil; 3 The Dental College of Georgia, Augusta University, Augusta, GA USA; 4 State Key Laboratory of Military Stomatology & National Clinical Research Center for Oral Diseases & Shaanxi Key Laboratory of Oral Diseases, Department of Prosthodontics, School of Stomatology, The Fourth Military Medical University, Xi’an, Shaanxi China

## Abstract

The present study evaluated the effect of different high-power-laser surface treatments on the bond strength between resin cement and disilicate ceramic. Lithium disilicate ceramic specimens with truncated cones shape were prepared and divided into 5 groups: HF (hydrofluoric acid-etching), Er:YAG laser + HF, Graphite + Er:YAG laser + HF, Nd:YAG laser + HF, and Graphite + Nd:YAG laser + HF. The treated ceramic surfaces were characterized with scanning electron microscopy and surface roughness measurement. Hourglasses-shaped ceramic- resin bond specimens were prepared, thermomechanically cycled and stressed to failure under tension. The results showed that for both the factors “laser” and “graphite”, statistically significant differences were observed (p < 0.05). Multiple-comparison tests performed on the “laser” factor were in the order: Er:YAG > Nd:YAG (p < 0.05), and on the “graphite” factor were in the order: graphite coating < without coating (p < 0.05). The Dunnett test showed that Er:YAG + HF had significantly higher tensile strength (p = 0.00). Higher surface roughness was achieved after Er:YAG laser treatment. Thus Er:YAG laser treatment produces higher bond strength to resin cement than other surface treatment protocols. Surface-coating with graphite does not improve bonding of the laser-treated lithium disilicate ceramic to resin cement.

## Introduction

There is an increasing trend for lithium disilicate ceramic to be used in restorative dentistry because of the esthetic values and high flexural strengths achieved by those restorations^1,2^. Different surface treatment techniques are used to enhance the bond strength between silicate-based ceramics and resin cements^3–5^. Hydrofluoric acid (HF)-etching is the most commonly used for conditioning silicate-based ceramic surfaces. Recent studies indicate that high-energy lasers such as Er:YAG (erbium-doped: yttrium aluminum garnet) and Nd:YAG (neodymium-doped: YAG) lasers further improve resin-ceramic bonding when laser treatment is used in conjunction with HF-etching. The Er:YAG laser removes surface particles by “ablation”, that involves micro-explosions and vaporization^6,7^. The Nd:YAG laser increases surface roughness to create better adhesion between the resin and ceramic surface^8,9^.

Laser irradiation creates micro-retentions that enhance the surface energy of the ceramic and facilitate silane application, resulting in more durable resin-ceramic bonding. This effect is related to pigmentation of the substrate and the amount of water it contains. Since, the ceramic substrate is almost water-free and its color is opaque white, a problem related to laser treatment is that the ceramic surface may not absorb the emitted energy sufficiently, thereby reducing the intended effects. Thus, a coating on the ceramic surface has been suggested as a means to increase laser energy absorption^10–13^. Graphite coating is often chosen for its high absorptivity qualities, ease of application, availability and also for economic reasons^10–13^.

Progress in adhesive dentistry has led to the development of different mechanical testing approaches to measure the bond strength between ceramic and resin cements. These approaches include tensile, microtensile, shear and microshear bond testing^3,4,10^. The purpose of the present study was to evaluate, using a different method of tensile strength testing, the bond strength between resin cement and lithium disilicate ceramic that had been subjected to different surface treatments, including the Nd:YAG laser and Er:YAG lasers, in the presence or absence of a graphite surface coating. The null hypothesis tested was that laser irradiation and graphite coating of the ceramic surface have no effect on the bond strength between resin cement and lithium disilicate ceramic.

## Materials and Methods

### Specimen preparation

One hundred and seventy lithium disilicate truncated cones (IPS e.max Press; Ivoclar-Vivadent, Schaan, Lichtenstein) were fabricated using a lost-wax technique. The low contraction wax (Renfert Geo; Renfert GmbH, Hilzingen, Germany) was poured into a 4-mm thick metal split-mold with a 2-mm diameter wide base and a 4-mm diameter wide top surface^14^. The specimens were heat-pressed according to the manufacturer’s instructions (Table 1).

**Table 1:** Brand names, manufacturers, chemical compositions and batch numbers of the materials used in the present study.

Brand names	Manufacturer	Chemical composition	Batch number
IPS e.max Press–LTD3	Ivoclar-Vivadent, Schaan, Liechtenstein	SiO_2_, Li_2_O, K_2_O, MgO, ZnO, Al_2_O_3_, P_2_O_5_ and other oxides	R36160
Condicionador de Porcelana	Condicionador de Porcelana, Dentsply DeTrey, GmbH, Germany	10% hydrofluoric acid	729554E
Variolink II base	Ivoclar-Vivadent	Bis-GMA, urethane dimethacrylate, triethylene glycol dimethacrylate. barium glass, ytterbium trifluoride, Ba-Al-fluorosilicate glass, spheroid mixed, oxide, catalysts, stabilizers, pigments	R43648
Variolink II catalyst	Ivoclar-Vivadent		R59556
Monobond Plus	Ivoclar-Vivadent	Alcohol solution of silane methacrylate, phosphoric acidmethacrylate and sulphide methacrylate	R71495

Abbreviations. Bis-GMA: bisphenol A-glycidyl methacrylate.

The pressed ceramic specimens were wet-polished with 600-grit silicon carbide paper in a circular polisher (DP-10; Panambra, São Paulo, SP, Brazil) using running water as coolant, and cleaned ultrasonically in distilled water for 5 min. The polished specimens were allocated to five experimental groups (N = 34; Table 1):

1. Control. Each specimen was etched with HF (60 sec), rinsed with water spray for 60 sec, silanized with Monobond Plus (Ivoclar-Vivadent) for 60 sec and air-dried.
2. Er:YAG laser. Each specimen was irradiated using a Er:YAG laser (Key Laser 3; KaVo Kerr, Washington, DC, USA) with 200 mJ energy, using a pulse repetition rate set at 10 pps, 2.94 µm wavelength and at 12 mm away from the specimen surface with water spray cooling (5 sec). The irradiated ceramic surfaces were then etched with HF (60 sec), rinsed with water spray (60 sec), silanized and air-dried.
3. Graphite + Er:YAG laser. Each surface was graphite-coated by rubbing graphite lead perpendicular to the surface prior to Er:YAG laser irradiation using the same parameters described in II. The graphite-and-laser-treated specimen was etched with HF (60 sec), rinsed with water spray (60 sec), silanized and air-dried.
4. Nd:YAG laser: Each specimen was irradiated using a Nd:YAG laser (PulseMaster 600 IQ; American Dental Technologies Inc., Corpus Christi, TX, USA) with 120 mJ energy. The pulse repetition rate was set at 15 pps and a 320 µm diameter laser optical fiber was placed in contact with the specimen surface for 1 min without water spray. The irradiated ceramic surfaces were then etched with HF (60 sec), rinsed with water spray (60 sec), silanized and air-dried.
5. Graphite + Nd:YAG laser: Each specimen surface was coated with graphite prior to Nd:YAG laser irradiation using the same parameters described in II. The graphite-and laser-treated-specimens were then etched with HF (60 sec), rinsed with water spray (60 sec), silanized and air-dried.

### Surface analysis

Two specimens from each group were employed for qualitative analysis of the treated surface. Each specimen was sputtered-coated with gold-palladium and examined using a scanning electron microscope (SEM; Inspect S50, FEI Company, Hillsboro, OR, USA) at different magnifications. Two additional specimens from each group were evaluated using a digital optical profilometer (Wyko NT 1100; Veeco, Plainview, NY, USA). Images were analyzed with the Vision v3.60 software (Veeco) for the arithmetic mean value of surface roughness (R_a_). Measurement of surface roughness was performed at a magnification of 20.5X on two areas of each specimen (center and periphery).

### Bonding procedures

**Figure 1:**
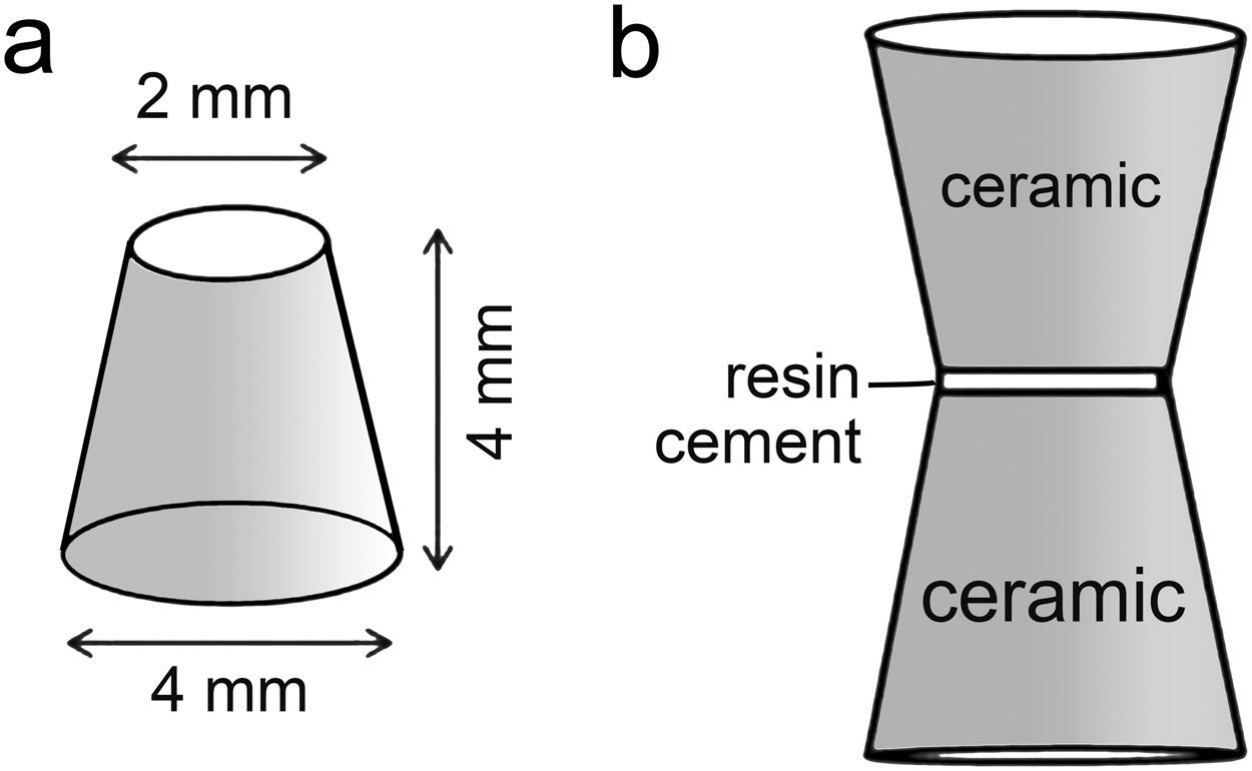
Specimen dimensions. (**a)** Lithium disilicate truncated cone. (**b**) Hourglass-shaped specimen.

The other 150 specimens (N = 15) were bonded (two truncated cones per bonding assembly) at the bases with a dual-cured resin cement (Variolink II; Ivoclar-Vivadent) (Fig. 1). The resin cement was light-cured for 40 sec using a light-emitting diode-type curing unit (Radii-Cal LED; SDI, Bayswater, Victoria, Australia).

### Thermomechanical aging

The bonded specimens were subjected to thermomechanical challenge that consisted of 24,000 mechanical cycles (30 N load at 4 Hz) and 1,000 thermal cycles (30 sec dwell-time in each water bath with temperatures maintained at 5 °C, 37 °C and 55 °C, respectively). Aging was conducted simultaneously in a thermomechanical cycler (Model ER-37000; ERIOS, São Paulo, Brazil).

### Tensile bond strength

**Figure 2:**
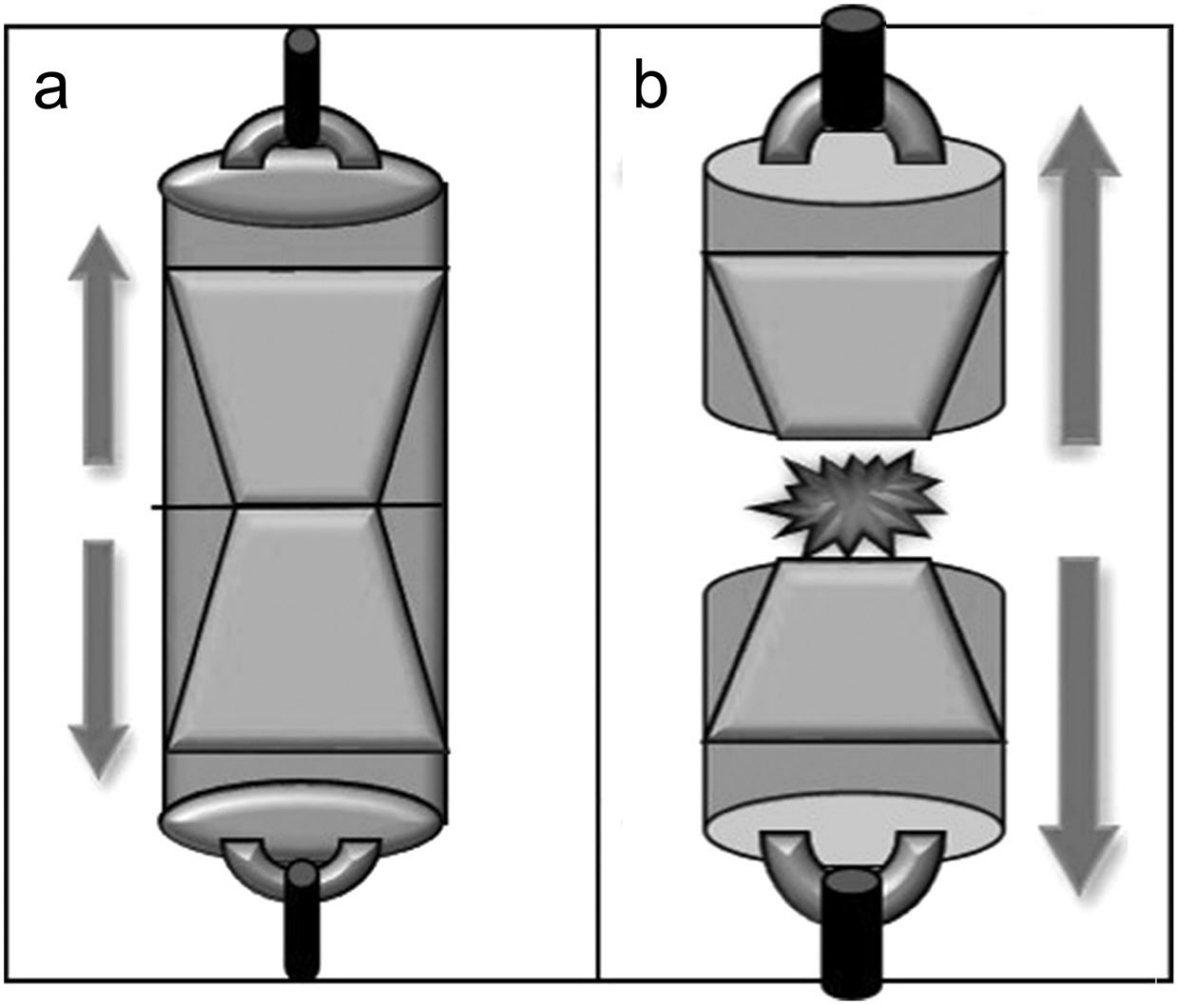
Schematic of the tensile test. (**a)** Before testing. (**b)** After stressing the bonded assembly to failure.

Bond strength was evaluated in a universal testing machine (model DI-1000; EMIC São José dos Pinhais, Brazil) by attaching a specimen to a custom-made device (Fig. 2), using a 10 kg load cell at a cross-head speed of 1 mm/min. Loading was performed in tension until failure. Results of tensile testing (in MPa) were statistically analyzed using one-factor analysis of variance (ANOVA) after affirming that the normality and equal variance assumptions of the data were not violated. The Dunnett test was employed for post-hoc comparison of the four experimental treatment groups with the HF control. In addition, two-factor ANOVA was used to examine the effects of “laser” and “graphite coating” on the tensile strength of resin cement to lithium disilicate ceramic (the control group was excluded from this analysis). Post-hoc pairwise comparisons were performed using the Tukey test. For all analyses, statistical significance was pre-set at α = 0.05. Two representative specimens from each group with bond strength close to the respective mean value were employed for qualitative analysis of the fractured interface. The specimens were sputtered-coated with gold-palladium and examined using SEM at different magnifications.

## Results

### SEM of pre-bonded, surface-treated specimens

**Figure 3:**
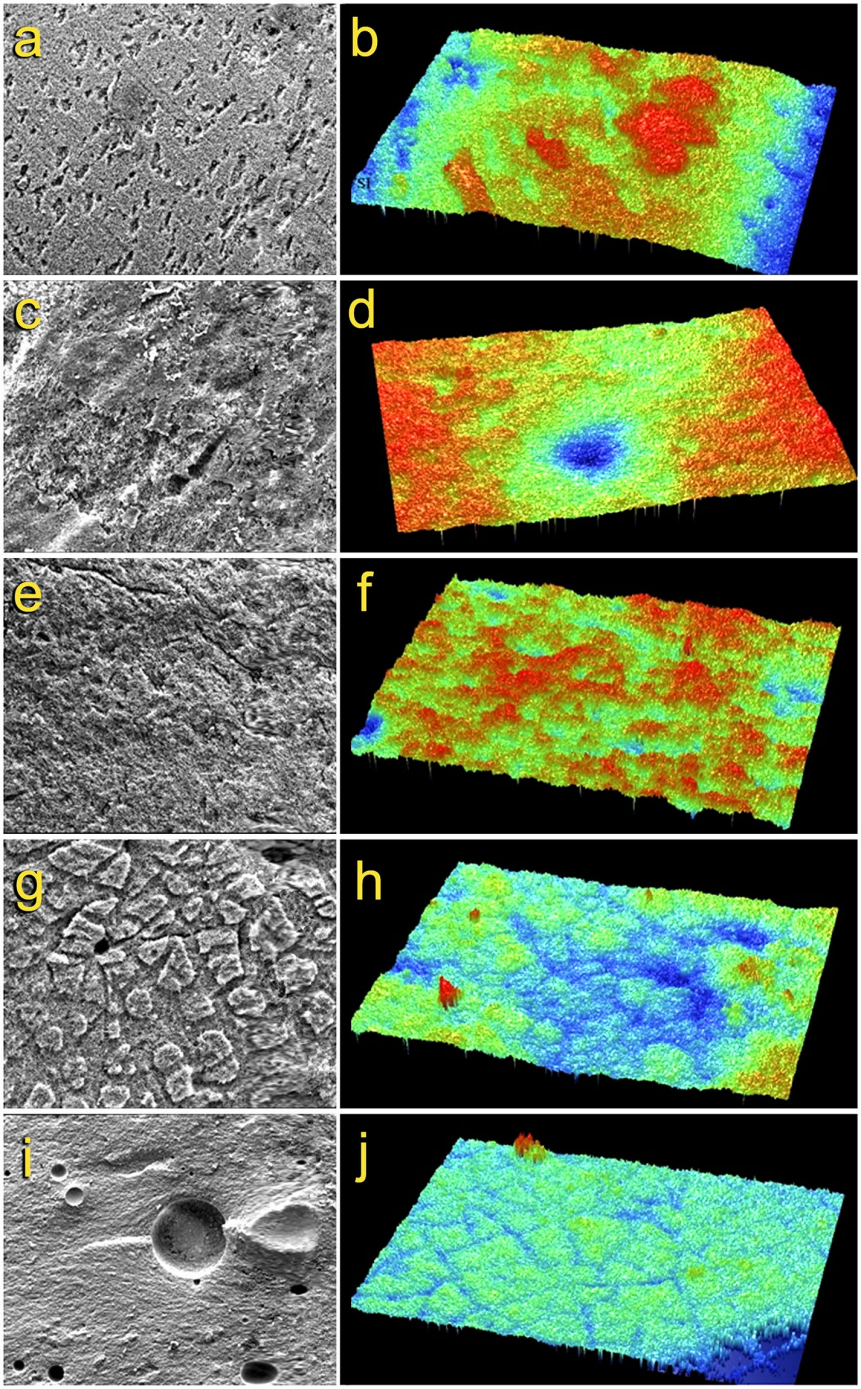
Scanning electron microscopy (SEM; left column) and 3-D profilometry images (20.5×; right column) of lithium disilicate specimens after different surface treatments. (**a)** SEM (1000×) and (**b**) profilometry images of the hydrofluoric acid-etched control group. (**c**) SEM (1000×) and (**d**) profilometry images of the Er:YAG laser group. (**e**) SEM (1000×) and (**f**) profilometry images of the Graphite + Er:YAG laser group. (**g**) SEM (1000×) and (**h**) profilometry images of the Nd:YAG laser group. (**i**) SEM (500×) and (**j**) profilometry images of the Graphite + Nd:YAG laser group. The lower SEM magnification enabled visualization of the craters scattered across the treated ceramic surface.

Scanning electron microscopy images (left column, Fig. 3) showed grooves and cracks in specimens that had been treated with Graphite + Er:YAG laser (Fig. 3e) and Nd:YAG laser treatments (Fig. 3g). The Graphite + Nd:YAG laser group (Fig. 3i) showed craters and surface modification in the ceramic when compared to all the other groups.

Roughness parameters (arithmetic means of all peaks and valleys) of the center and periphery of the surface-treated specimens are reported in Table 2. Higher surface roughness was achieved after the lithium disilicate specimens were subjected to Er:YAG laser treatment (Fig. 3d), while lower surface roughness was seen after specimens received Graphite + Nd:YAG laser treatment (Fig. 3j). The profilometry images (right column, Fig. 3) complemented what the SEM observations.

**Table 2:** Mean (standard deviations) tensile bond strength values (in MPa) and surface roughness results (R_a_, in µm) for the control and experimental groups.

Groups	Means (standard deviation)	Minimum	Maximum	Roughness Surface (center)	Roughness Surface (periphery)
Control	14.3 (4.4)	8.2	22.18	2.64	1.39
Er:YAG laser	27.5 (7.1)	8.9	32.7	3.48	2.14
Graphite + Er:YAG laser	12.1 (3.5)	8.4	18.8	1.29	1.16
Nd:YAG laser	17.7 (4.9)	11.8	17.4	2.69	1.46
Graphite + Nd:YAG laser	7. 7 (3.6)	3.1	15.5	0.91	0.86

### Tensile bond strength

Descriptive statistics (means and standard deviations) of the tensile testing results are shown in Table 2. There was no pre-test failure when the hourglass-shaped bonded specimen configuration was employed.

Group 2 (Er:YAG laser) showed the highest tensile strength (27.5 ± 7.1 MPa). Graphite coating reduced the tensile strengths of the two laser-irradiated groups, especially for Group V (Graphite + Nd:YAG laser). The Dunnett test was used to compare the Control group with the other four experimental groups. Only the tensile strength of Group 2 (Er:YAG laser) was statistically different (p = 0.00002) from the Control (Group 1) (Table 3).

**Table 3:** Dunnett test comparing the laser-irradiated and graphite-coated groups with the control group.

Compared with Control group	p Value
Er:YAG laser	0.00002^*^
Graphite + Er:YAG laser	0.602
Nd:YAG laser	0.076
Graphite + Nd:YAG laser	0.999

^*^Significantly different (p < 0.05).

The effects of Nd:YAG/Er:YAG laser irradiation, and the presence/absence of graphite coating on the bond strength between lithium disilicate and resin cement were analyzed by two-way ANOVA. Both the factors “laser” (p = 0.00) and “graphite coating” (p = 0.00) significantly affected tensile strength of the resin cement to the lithium disilicate ceramic. The interaction of thoese two factors was not statistically significant (p = 0.059). Post-hoc Tukey test showed that Er:YAG laser treatment (mean value: 21.7 MPa) produced higher tensile bond strength (p < 0.05) than Nd:YAG laser treatment (mean value: 12.9 MPa), while groups with graphite coating (mean value: 11.5 MPa) produced lower tensile strength (p < 0.05) when compared to the groups without graphite coating (mean value: 23.8 MPa).

### Analysis of fractured interface

Microscope fracture analysis showed that most of the failures were adhesive and cohesive within the cement for all groups except for the Graphite + Nd:YAG laser group, in which only adhesive failure was observed.

## Discussion

The results of the present study indicate that the adjunctive use of laser surface treatment improves the tensile strength of resin cement to lithium disilicate, while pre-coating the ceramic surface with graphite prior to laser irradiation and HF etching adversely affects the tensile strength between the cement and ceramic. Thus, the null hypothesis that laser irradiation and graphite coating of the ceramic surface have no effect on the bond strength between resin cement and lithium disilicate ceramic has to be rejected. Thermomechanical cycling was employed as a form of artificial aging in the present study to incorporate an element of durability into the testing of the resin-ceramic bonds. There was no comparison of tensile strengths before and after aging because the study objective was to evaluate differences in bond strength among different surface treatments after aging.

Previous studies evaluated resin-ceramic bond strength using shear, micro-shear or micro-tensile testing techniques^3,4,10,12^. Stress induction during specimen preparation for mechanical testing, as well as the different shapes and geometry of the prepared specimens may induce interference to the testing results, leading researchers to discordant conclusions. In their design of the microtensile test, Sano *et al*. opined that the tensile strength is inversely proportional to the size of tested surface, and that adhesive failure predominantly occurs if the cross-sectional surface area is approximately 1 mm^2,15,16^. However, the “stick” or “beam” version of the microtensile test is not a practical test for evaluating bonding to ceramics. This is because stress induction during specimen results in many premature failures prior to testing^17^. The ‘trimmed’ version of the microtensile test requires the researcher to prepare hourglass-shaped specimens^18^. When trimming is not carefully performed, interfacial defects may easily be introduced that precipitates crack propagation during tensile loading of the specimen, eventually resulting in premature failure and much reduced interfacial strength. The shear test is another simple mechanical test to perform, but there is difficulty in placing the specimen in a defined area and maintaining that position during loading, which results in a lot of friction^19^. Both the micro-tensile and shear tests may generate unrealistic results. Because of the difficulty in obtaining realistic bond strength data between resin cements and ceramics, the present study adopted a method that combines the advantages of tensile and micro-tensile tests by using small bond surface diameter (2 mm) and avoiding stress induction during specimen preparation. The test yielded acceptable results with no premature failures.

Many studies have reported ways to improve the bond strength between resin cement and ceramics by creating micro-retentions or other modifications on the ceramic surface^4,7,20–23^. The present study shows that Er:YAG laser irradiation followed by HF etching increases the bond strength of lithium disilicate ceramics to resin cement when compared to the control group (Table 3). These findings are contrary to those reported by Foxton *et al*.^24^ and Akyil *et al*.^25^. The difference may be explained by the method employed for mechanical testing. Foxton *et al*.^24^ employed the shear test while Akyil *et al*.^25^ used the micro-tensile test to evaluate the strength of the resin-ceramic bond. The Er:YAG laser irradiation parameters employed were also different. Different power settings have been employed in the literature for laser irradiation of ceramic surfaces^6,7,9,10,12,24,26,27^. The power setting adopted in the present study was based on the one used by Cavalcanti *et al*.^12^, which involved a lower power setting (220 mJ) and constant water cooling.

The Nd:YAG laser surface treatment demonstrated worse results than the Er:YAG laser group and the results were not significantly different from the HF control group. When specimens were analyzed for surface roughness, those derived from the Er:YAG laser group had higher R_a_ values, followed by specimens from the Nd:YAG laser group (Table 2). These findings were different from those reported by Kara *et al*.^26^, who concluded that there was no significant difference between Nd:YAG laser and Er:YAG laser irradiation on the roughness of lithium disilicate surfaces.

Studies employing surface coating with graphite prior to laser irradiation suggested that such a coating increases energy absorption, which in turn, improves the efficacy of ceramic surface modification by the laser^4,12,13,24^. Results derived from the present study showed that placement of a graphite coating adversely affected the efficacy of both lasers. The lower surface roughness that resulted from the presence of a graphite coating was complementary to the tensile testing results. In the future, additional studies are necessary to investigate the efficacy of the graphite coating technique, because all of the previous studies evaluated laser irradiation with graphite coating in all groups.

Scanning electron microscopy showed that the use of Er:YAG laser irradiation followed by HF etching modified the lithium disilicate surface. It appears that the period of laser application (5 sec) has to be extended, because morphologic examiniation revealed some locations on the laser-treated surface that were similar to the control group. The Graphite + Er:YAG laser and Nd:YAG laser groups showed reduction of surface roughness; however, the micro-structures were not modified and appeared similar to the HF control group. Nevertheless, more research comparing different resin cements is warranted.

## Conclusions

Within the limits of the study, the following conclusions may be drawn:

1. The hourglass-shaped specimen design is effective for measuring resin-ceramic bond strength under tension by completely eliminating pre-test failure.
2. The Er:YAG laser produces higher bond strength than the other surface treatment regimens when it is used adjunctively with hydrofluoric acid. Such a technique may be considered during bonding of resin cement to lithium disilicate ceramic.
3. Surface coating with graphite prior to laser irradiation does not increase in bond strength between the resin cement and ceramic.
